# Synthesis, Application and Prospects of Carbon Dots as A Medicine Food Homology

**DOI:** 10.3390/nano15120906

**Published:** 2025-06-11

**Authors:** Siqi Huang, Huili Ren, Hongyue Chen, Nuan Wen, Libo Du, Chaoyu Song, Yuguang Lv

**Affiliations:** 1 School of Pharmacy, Jiamusi University, Jiamusi 154002, China; 15663050135@163.com (S.H.); 15156027890@163.com (H.R.); chenhongyue2023@163.com (H.C.); 2 The First Affiliated Hospital of Jiamusi University, Jiamusi 154002, China; 3 Center for Molecular Sciences, State Key Laboratory for Structural Chemistry of Unstable and Stable Species, Institute of Chemistry, Chinese Academy of Sciences, Beijing 100000, China; 4 School of Chemistry and Chemical Engineering, Shanghai Jiao Tong University, Shanghai 200000, China; songchaoyu@sjtu.edu.cn

**Keywords:** carbon dots, medicine food homology, detection of pollutants, biomedical science, biological imaging, biological sensing, nanocatalysis

## Abstract

Against the background of the vigorous development of materials science and the deep cross-infiltration in many fields, a new medicine food homology, carbon dots (herein combined and abbreviated as MFH-CDs), has sprung up, showing great potential. This review used ChatGPT 4.0 to collect background information related to carbon dots, focusing on the common rich medicinal and food resources such as *Lycium barbarum*, Chinese yam, honeysuckle, and *Ganoderma lucidum*. These carbon dots are synthesized by hydrothermal synthesis, microwave radiation, and pyrolysis, which have the advantages of small particle size, high quantum yield, and low cytotoxicity. Recent studies have found that MFH-CDs have great application potential in biosensors, biological imaging, and drug delivery. In this paper, the characteristics of preparing carbon dots from different medicinal and edible resources and their applications in biology in recent years are reviewed, which provides in-depth guidance for the research and application of carbon dots from medicinal and edible biomass, helps it shine in multidisciplinary fields, and opens a brand-new journey from traditional medicinal and edible culture to cutting-edge technology application.

## 1. Introduction

“Nanomaterials” was first proposed by Gleiter in 1983, which refers to materials with at least one dimension in the range of 1–100 nm in a three-dimensional space [1]. When the particle size is reduced to the nanometer scale, it will lead to different characteristics of acoustic, optical, electrical, magnetic, and thermal properties, which will make it have greater optical properties and stronger mechanical properties. Among the many nanomaterials, carbon nanoparticles have attracted much attention.

The external shapes of carbon nanoparticles are diverse, with common shapes including spherical, ellipsoidal, and rod-like shapes. Just like various small objects in our daily lives, they can be round, oval, or elongated. However, carbon nanoparticles are extremely small, so small that they cannot be seen with the naked eye and require special microscopes to be observed. Their sizes are typically on the nanoscale, with 1 nanometer equal to one billionth of a meter. The internal structure of carbon nanoparticles resembles a miniature “cage” or “honeycomb” constructed from carbon atoms. Carbon atoms are interconnected through strong chemical bonds, forming a stable structure. These carbon atoms are arranged very tightly, like a sturdy model built with building blocks. Inside this “cage” or “honeycomb,” there may be some voids that can accommodate other substances, just like small boxes can hold things. This is due to the arrangement of atoms and growth conditions during the formation of carbon nanoparticles, which lead to the formation of internal pores [2]. For example, in certain preparation methods, carbon atoms grow around specific templates or impurities. After the templates or impurities are removed, these voids are left behind [3,4]. The surface of carbon nanoparticles is not smooth, but it has many tiny protrusions, depressions, or wrinkles. This is similar to the surface of an orange. This unique structure of the carbon nanoparticles’ surface results in a relatively large surface area. The large surface area allows carbon nanoparticles to better contact and react with other substances. This unique structure provides advantages for carbon nanoparticles in terms of contact and reaction with substances, thereby greatly promoting the in-depth exploration of carbon nanoparticles.

In 2004, Xu et al. [5] first reported and described it as “fluorescent nanoparticles” in the process of preparing single-walled carbon nanotubes by electrophoresis. The generation of fluorescence originates from the electronic transitions of molecules. Electrons in molecules exist in different energy levels. In the ground state, they are in low-energy orbitals. When a molecule absorbs photons of a specific wavelength, the electrons transition to an excited state. Since the excited state is unstable, the electrons will return to the ground state through radiative transitions and emit fluorescent photons. Molecules with chromophore structures such as conjugated double bonds and aromatic rings are more likely to produce fluorescence [6]. In the field of pharmacology, fluorescent substances can be used to label biomolecules. For example, fluorescent dyes can be linked to antibodies, and then through fluorescence imaging, specific biomolecules can be traced in the living body to assist in disease diagnosis. Additionally, by observing the changes in the fluorescence properties of fluorescently labeled ligands after they bind to biological targets, the mechanisms of drug action can be explored [7,8]. In physical chemistry, different fluorescent substances have unique excitation and emission spectra, based on which substances can be qualitatively and quantitatively analyzed. Moreover, the interaction between some substances and fluorescent molecules can cause fluorescence quenching. Based on this, fluorescent sensors can be designed to detect specific substances or environmental parameters, such as oxygen concentration. All these applications are based on the molecular basis of fluorescence and the characteristics it exhibits when interacting with other substances, which endows fluorescence with extensive applications and significant value in multiple fields [9].

However, it was not until 2006 that Sun et al. [10] formally defined “carbon dots” (CDs) as spherical and monodisperse nanoparticles with a particle size less than 10 nm, and carbon dots refer to new nanomaterials discovered after carbon nanotubes, fullerenes, and graphene [11,12,13]. Since then, there have been sporadic reports on the nature of CDs, but the basic knowledge about CDs is still elusive. Researchers mainly focus on the refined improvement of preparation methods and the diversified expansion of application scenarios. In the search for synthetic raw materials, researchers have invested a lot of energy in chemical substances and various natural substances. However, the research on synthesizing carbon dots with medicinal and edible homologous substances as carbon sources and exploring their applications is relatively scarce.

**Figure 1:**
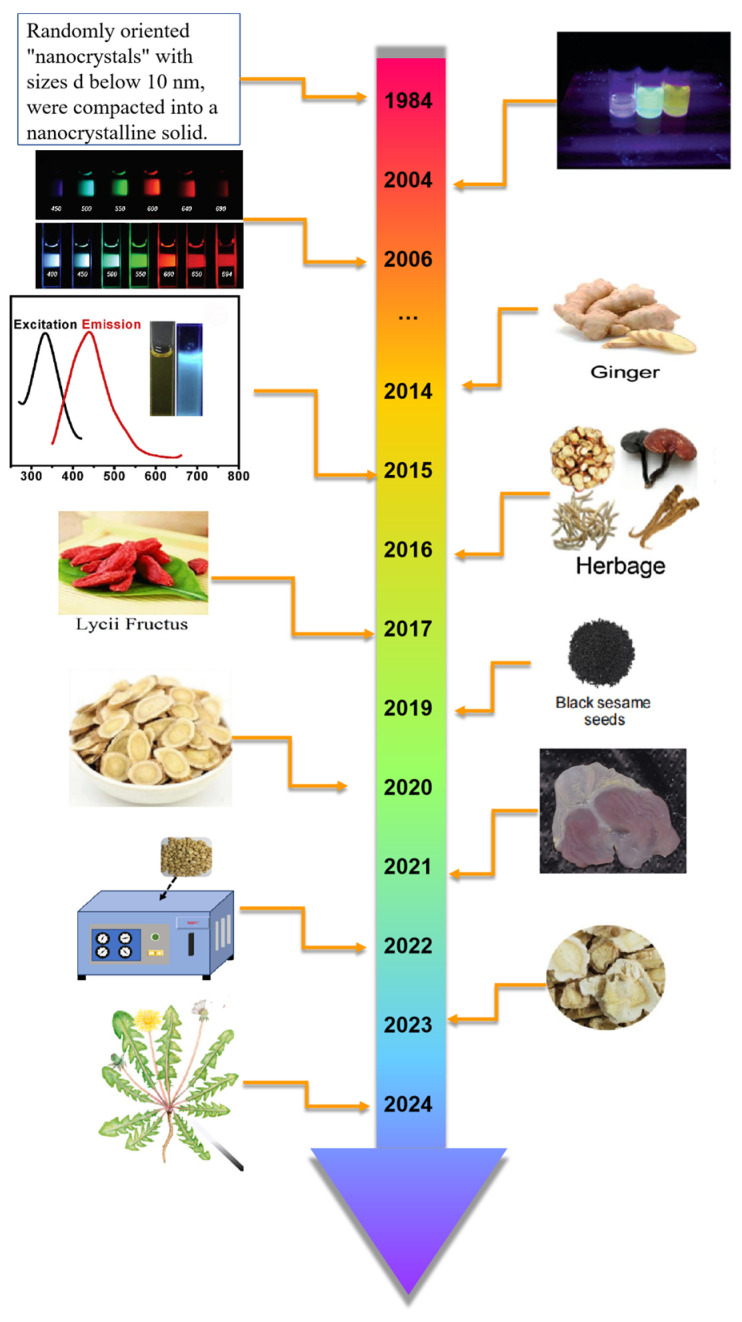
Short timetable for the development of CDS and MFH-CDs (in chronological order [1,5,10,18,19,20,21,22,23,24,25,26,27]).

**Figure 2:**
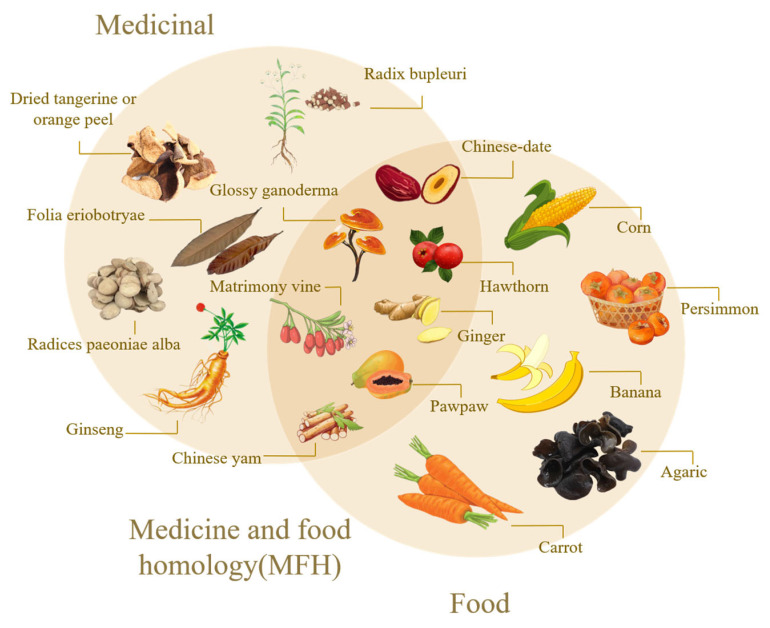
A homologous relationship between mate-ria medica and food.

With the development of society, the production concept of green chemistry has become deeply rooted in people’s hearts, and taking “green” biomass as a raw material has gradually become a hot spot in production research. Especially in the synthesis of precursors, a large number of green precursors can be used as natural carbon sources for the synthesis of CDs, which has obvious advantages such as rich and renewable raw materials, no chemical pollution, and environmental friendliness [14]. Various green carbon precursors have been studied and applied, including fruits, vegetables, and various foods and beverages [15], in order to realize the synthesis of materials with good material characteristics, high cost efficiency and economic and environmental protection. Among these green precursors, the homology of medicine and food (MFH) [16] has attracted wide attention because of its unique medicinal value. MFH refers to many foods, that is, medicines. In traditional medicine, some substances can be used as part of a daily diet to maintain the normal physiological functions of the human body, and they can also play a role in certain medical treatments of specific diseases, which embodies the wisdom concept of China’s traditional health care and disease prevention. Medicinal food varieties developed according to MFH theory have the function of preventing and treating chronic diseases and protecting health. Figure 1 shows the development history of CDS and MFH-CDs. By 2024, the National Health Commission has published more than 110 kinds of MFH Chinese herbal medicines, such as *Lycium barbarum*, Chinese yam, hawthorn, etc. [17]. Figure 2 shows the homologous relationship between Chinese herbal medicines and foods. After long-term practice, these substances have been confirmed to have good safety and certain health care and medicinal values.

Medicinal and edible foods are rich in bioactive components such as flavonoids, saponins, alkaloids, and polysaccharides. These components may participate in the surface modification of carbon dots or form complexes with carbon dots during the synthesis process, endowing the carbon dots with special properties. Taking hawthorn as an example, the flavonoids in it may combine with carbon dots during the synthesis of hawthorn carbon dots, endowing them with certain biological activities such as antioxidant and hemostatic properties. Tian et al. [28] investigated the material basis and mechanism of action of hawthorn carbon’s hemostatic effect from the perspectives of chemical composition and carbon dots. The study found that the separation of conifer aldehyde from hawthorn charcoal can significantly reduce thrombin time, increase fibrinogen content, and enhance platelet aggregation, thereby exerting a hemostatic effect. Further separation and purification yielded multifunctional hawthorn carbon dots (CFCDs) with a particle size of 2.35 ± 0.67 nm, in a graphite crystalline form. The surface contains abundant hydroxyl and carboxyl functional groups, exhibiting high water solubility and good stability, and demonstrating hemostatic properties. This study preliminarily clarifies the material basis for the hemostatic effect of hawthorn carbon and the mechanism of hawthorn carbon’s hemostatic action, providing a theoretical basis for the clinical application of hawthorn carbon. It also offers valuable references for the research of other carbon-based medicines and the discovery of multifunctional carbon dots from natural materials.

Due to the differences in the types and content of active ingredients in MFH substances, the biocompatibility of the derived carbon dots also exhibits diverse characteristics. Specifically, carbon dots derived from MFH are usually composed of elements such as carbon, hydrogen, oxygen, and nitrogen, and their surfaces may contain abundant functional groups such as hydroxyl, carboxyl, and amino groups. These functional groups endow the carbon dots with good water solubility and biocompatibility. In terms of physicochemical properties, MFH-derived carbon dots typically have nanoscale dimensions, excellent optical properties (such as fluorescence emission), good thermal stability, and chemical stability. These characteristics make them highly applicable in the fields of biomedicine and materials science. For example, the bioactivities of carbon dots derived from MFH vary from one another, with most exhibiting pharmacological effects such as antibacterial [22,23], antiviral [24], antitumor [25], free radical scavenging [26], and anti-gout [14,27]. These diverse bioactivities are closely related to their unique physicochemical properties, further expanding their application prospects in the fields of medicine and functional materials. Moreover, with their excellent photoluminescence properties [15], low toxicity, good water solubility [16], and high biocompatibility, they can also be used in fields such as ion detection [17,18,29,30], drug molecule sensing [31,32,33], cell imaging [19,34,35,36], drug delivery [37,38,39], and photocatalysis [39,40]. Therefore, selecting the appropriate MFH as a carbon source to synthesize carbon dots with fluorescence stability and pharmacological activities has certain guiding significance for exploring the inherent biological activities, material compositions, and physical and chemical properties of the carbon dots derived from MFH.

## 2. Materials and Methods

When writing this manuscript, we used ChatGPT 4.0 to collect background information related to carbon dots. The specific steps are as follows:

Information collection on carbon dot synthesis methods: Using ChatGPT to search for carbon dot synthesis methods can quickly provide a summary of various synthesis pathways. Based on these preliminary results, further manual searches of specialized databases were conducted to find detailed research literature on each synthesis method. By reviewing and analyzing these documents, key information was obtained, which was then used to construct and list Table 1. Table 1 clearly shows the synthesis methods, applications, and comparison parameters of the different carbon dots.

MFH-CDs Application Information Collection: Similarly, using ChatGPT, we explored the application fields of MFH-derived carbon dots (MFH-CDs). ChatGPT provided suggestions for potential applications of MFH-CDs in areas such as bioimaging, sensor detection, and catalysis. Subsequently, using this as a clue, we delved into authoritative academic databases such as Web of Science to search for the latest cutting-edge literature. We carefully read and summarize these documents, extracting key content such as the mechanisms of action, performance advantages, and practical application cases of MFH-CDs in various fields. These details are then elaborated upon in the manuscript, along with specific application examples, providing direct references for related research.

Explanation of Insufficient Search and Solutions: Although ChatGPT played an important role in the initial stage of information gathering, it also has certain limitations. For example, the information it provides may have issues such as being outdated or containing inaccuracies in some literature citations. To this end, in the summary and outlook section, we return to professional academic databases, extensively review the relevant literature, and identify the specific shortcomings encountered when using ChatGPT to search for carbon dot information. To address these shortcomings, corresponding solutions have been proposed. For the issue of inaccurate citations, each piece of the literature provided by ChatGPT is cross-verified, and its accuracy is checked against multiple databases to ensure the reliability and scientific validity of the information on which the manuscript is based.

**Figure 3:**
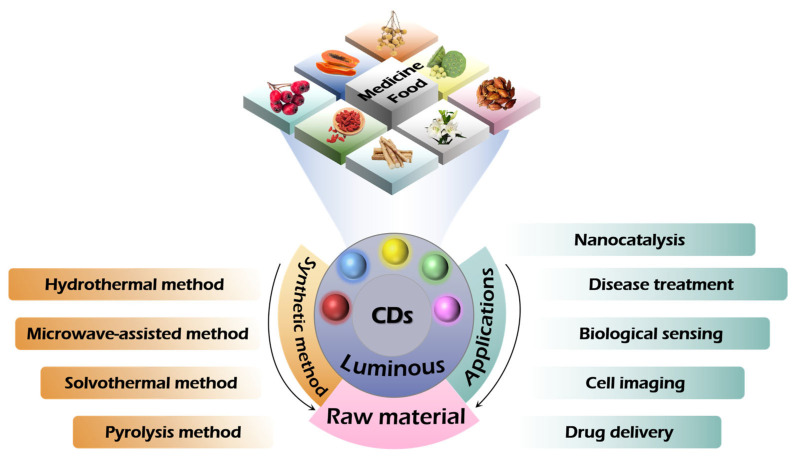
Synthetic characteristics and application exploration of homology carbon dots in medicine and food.

Figure 3 focuses on medicinal and edible carbon dots (MFH-CDs), clearly displaying information related to their synthesis methods and applications. In the synthesis section, various synthesis methods are employed to demonstrate the diversity of raw materials and the multiplicity of methods. By showcasing examples of its application in various fields, highlight the significant value of MFH-CDs in multiple domains such as biomedicine and environmental monitoring.

## 3. Synthesis Method of MFH-CDs

**Figure 4:**
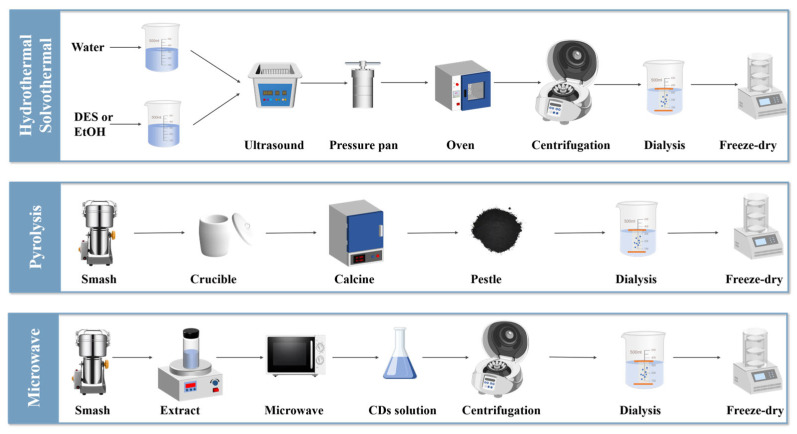
Roadmaps of different synthesis methods.

From the perspective of green chemistry, the preparation of carbon dots (CDs) from natural products by various synthetic methods has become a research direction that attracts much attention. However, MFH-derived carbon dots (MFH-CDs) also face the issue of low synthesis yield in practical applications, similar to CDs. On one hand, synthesis methods such as the commonly used hydrothermal method and the microwave-assisted method, although easy to operate, can result in unstable carbon dot formation efficiency due to slight changes in reaction conditions (such as temperature, time, and precursor concentration), making it difficult to ensure high yield. On the other hand, the yield of MFH-CDs is also influenced by the precursor materials. The composition of food-medicine homologous substances is complex, and during the carbonization process, some effective components may decompose or undergo side reactions, preventing them from fully converting into carbon dots, thereby reducing the yield. Nevertheless, by optimizing reaction conditions, improving synthesis processes, and selecting suitable precursor combinations, the synthesis yield of MFH-CDs is expected to be enhanced, providing stronger feasibility and economic support for their widespread application in fields such as biomedicine and materials science. For a long time, Chinese researchers have carried out in-depth and extensive exploration on the preparation of MFH-CDs and have made a series of remarkable and important achievements in the quality control and application expansion of CDs synthesis. In the field of synthesis methods of CDs, its controllable synthesis is still in the initial stage of development, which can be divided into two main categories: “top-down” and “bottom-up”. The “top-down” method is usually achieved by disassembling carbon materials by physical or chemical means, while the “bottom-up” method involves the pyrolysis or carbonization process of small molecular organics. When using the “top-down” method to synthesize CDs, harsh experimental conditions (such as strong acid environment and arc discharge, etc.), complicated operation steps, and expensive instruments and equipment are often needed, which greatly restricts its practical application scope. Therefore, considering the relatively simple preparation process and cost-effectiveness factors, the “bottom-up” approach with MFH as the precursor has become an efficient method to obtain high-quality CDs. At present, the common “bottom-up” synthesis methods (Figure 4) include the hydrothermal method [17,21,22,23,41,42,43,44,45,46,47,48,49,50,51,52,53,54,55,56,57,58,59,60,61], the pyrolysis method [24,26,32,68,69,70,71], the solvothermal method [64,65,66,67], and the microwave-assisted method [27,29,62,63]. Supchocksoonthorn et al. [22] synthesized carbon dots from black sesame using a hydrothermal method and used them as a dual-mode probe for detecting ammonia in both gas and solution phases. The size of the carbon dots is approximately 7.6 nm, and they exhibit blue emission under UV excitation in solution, with a quantum yield of 2%. Long et al. [27] synthesized high quantum yield tricolor CDs from the natural precursor dandelion through a one-step solvothermal treatment. Structural and optical characterization indicates that the CDs possess excellent photostability, salt resistance, and thermal stability, as well as high quantum yields, with quantum yields of 43.8%, 32.9%, and 48.1% for B-CD, G-CD, and O-CD, respectively. In the bottom-up synthesis of MFH-CDs, fluorescent impurities are usually produced, so it is necessary to improve product purity by optimizing synthesis conditions and purification methods. By precisely controlling the reaction temperature, time, and raw material ratios, side reactions can be reduced, and impurity formation can be minimized. In terms of purification, dialysis can remove small molecular impurities, while centrifugation and column chromatography techniques can further separate high-purity MFH-CDs to meet research and application needs [72,73]. The detailed comparison of these synthesis methods is summarized in Table 1.

### 3.1. Hydrothermal Method

The hydrothermal method is one of the most commonly used methods to prepare MFH-derived carbon dots (CDs) [74]. In this method, distilled water is used as a conventional solvent and a reaction medium, and the raw materials are added into a stainless steel autoclave with a PTFE lining and heated continuously for a certain period of time in a specific temperature range (80 °C to 300 °C) and a high-pressure environment (generated by the reaction system itself), thus preparing the target product CDs [75]. Generally speaking, the surface of CDs synthesized by hydrothermal method does not need additional passivation treatment, which greatly improves the safety of the product and effectively reduces the potential toxicity risk. This is because the hydrothermal method has the advantages of simple operation and low cost and can realize the heterogeneous reaction in a reaction system (namely “one-pot method”), so it has been widely concerned and applied in related research fields.

Ginger (*Zingiber officinale* Roscoe) is the fresh rhizome of *Zingiber officinale*, a perennial herb of Zingiberaceae, which is pungent and mild in nature. It can enter the lungs, spleen, and stomach meridians. It has the effects of relieving exterior syndrome, dispelling cold and warming, stopping vomiting, warming the lungs and relieving coughs, and removing toxic substances. Fluorescence carbon dots (CDs) extracted by Li et al. [25] for the first time and obtained at 300 °C hydrothermal condition was used for cell imaging. At the same time, it is proved by experiments that the synthesized CDs can effectively inhibit the growth of human liver cancer cells.

Yam (*Dioscorea opposita* Thunb.) is the dry rhizome of *Dioscorea opposita*, which helps digestion, and functions as a hypoglycemic and an antioxidant. Chen et al. [43] synthesized YAM-CDs by hydrothermal method with yam as a precursor. The average size of YAM-CDs is 2–6 nm. The quantum yield is 4.44%, and it has good fluorescence stability and biological safety.

Hawthorn (*Crataegus pinnatifida*) belongs to the spleen, stomach, and liver meridian, and it has the effects of promoting digestion, invigorating the stomach, promoting qi circulation, removing blood stasis, removing turbidity, and lowering blood fat. Zhang et al. [44] used hawthorn as a carbon source and diethylenetriamine (DETA) as a nitrogen source to synthesize an environmentally friendly nitrogen-doped carbon dot (N-CDs) by using a one-pot hydrothermal method and established a fluorescent sensor for the rapid detection of chlortetracycline in pork samples. The quantum yield of N-CDs is 22.96%, the emission wavelength is 447 nm at the maximum excitation wavelength of 370 nm, and the average particle size is 3.17 nm.

Goji berry (*Lycium chinense* Miller) belongs to the liver, kidney, and lung meridian, and has the effects of nourishing the liver and kidneys, benefiting eyesight, and moistening the lungs. Tang et al. [38] successfully transformed *Lycium barbarum* into beneficial green luminescent (527 nm) fluorescent nitrogen-doped carbon dots (N-CDs) by hydrothermal synthesis for the first time. N-CDs have an extremely stable green fluorescence and quantum yield as high as 21.8%.

Jinhua bergamot (*Citrus Bergamot*) belongs to the liver, spleen, and stomach meridian, which has the effects of soothing the liver and regulating qi, harmonizing the stomach, and relieving pain, eliminating dampness, and resolving phlegm. Yu et al. [19] synthesized green CDs with Jinhua bergamot as a precursor by using the hydrothermal method at 200 °C for 5 h, and the quantum yield (QY) was as high as 50.78%.

### 3.2. Solvothermal Method

According to the above, a large number of studies show that MFH-CDs are usually prepared by using the hydrothermal method with water as the reaction solvent. By changing the kinds of reaction solvents, the PL properties of CDs can be effectively adjusted, and the multicolor emission of CDs can be realized [76]. The operation flow of the solvothermal synthesis method is to put the carbon source and one or more solvents in a stainless steel autoclave with Teflon lining. After heating for a certain period of time, the original carbon source will be transformed in the air atmosphere or inert atmosphere under the environment of high temperature and high pressure, and finally CDs will be generated. There are many similarities between the solvothermal method and the hydrothermal method, such as environmental friendliness, low cost, convenient operation, and simple equipment. Moreover, in the process of synthesizing CDs with the solvothermal method, the types of carbon precursors can be varied, which provides more choices and possibilities for the preparation of CDs. The use of organic solvents in the synthesis of CDs promotes the carbonization process, which leads to great changes in the photophysical properties of carbon nanoparticles.

Dangshen (*Codonopsis Radix*) is sweet and flat, and enters the spleen and lung meridian. With the functions of invigorating the spleen, benefiting the lung, nourishing the blood, and promoting fluid production, Qiu et al. [77] used a one-step solvent method to prepare green CDs from *codonopsis pilosula* at room temperature. Compared with the hydrothermal method, this method does not need expensive heating equipment and a long-term high-temperature heating process, and it has obvious advantages such as more convenient operation, lower energy consumption and more economical synthesis cost. The obtained *Codonopsis pilosula*-derived CDs (CP-CDs) have excellent fluorescence performance (QY is as high as 12.8%) and high light stability, and the surface of CP-CDs does not need any passivation or functionalization treatment.

### 3.3. Pyrolysis Method

The steps of synthesizing MFH-CDs by pyrolysis are similar to the processing of traditional Chinese medicine. In this process, the organic matter in the carbon source undergoes high temperature in a vacuum or inert atmosphere and is gradually transformed into carbon dots through heating, dehydration, degradation, and carbonization. In order to decompose carbon precursors into carbon nanoparticles, high concentrations of acids or bases are often used. Excessive temperature will lead to excessive oxidation of the carbon source, and the surface structure of carbon dots may be damaged, which will lead to the decline of its optical properties. The effect of reaction time on pyrolysis is very similar to that of the reaction temperature. Too short a reaction time will lead to insufficient carbonization of the carbon source, while too long a reaction time may lead to excessive carbonization and damage to the surface structure of carbon dots. Therefore, by changing the pyrolysis conditions, such as pyrolysis temperature and pyrolysis time, the properties of the obtained carbon dots can be adjusted. When preparing carbon dots through pyrolysis, it is common to produce some gaseous byproducts, such as carbon dioxide, carbon monoxide, and methane. However, during the pyrolysis process, if organic materials undergo incomplete oxidation reactions, there is a possibility of generating formaldehyde. Formaldehyde, among aldehyde substances, is a known carcinogen. When carbohydrates, proteins, and other components in medicinal food homology substances decompose and polymerize under pyrolytic conditions, carcinogenic substances like formaldehyde may be produced. In incompletely pyrolyzed macromolecular organic matter, there may also exist certain polycyclic aromatic hydrocarbons that are carcinogenic. Polycyclic aromatic hydrocarbons are typically produced from the incomplete pyrolysis of organic substances in a high-temperature, oxygen-deficient environment. If the reaction conditions are not properly controlled and the reaction is not sufficiently completed, polycyclic aromatic hydrocarbons may be generated, posing a potential threat to human health. To avoid the production of carcinogenic substances during the preparation of carbon dots, advanced pyrolysis reactors can be employed, such as rotary kiln furnaces, which ensure more uniform heating, reduce localized overheating or incomplete reactions, and decrease the likelihood of carcinogenic substance generation [78].

Chinese bellflower (*Platycodon grandiflorus*) can treat coughs with excessive phlegm and chest tightness. Chen et al. [26] successfully synthesized PGC-CDs with a diameter of 1.2–3.6 nm by heating *Platycodon grandiflorum* at 350 °C for 1 h. The experimental results show that PGC-CDs can effectively inhibit the level of inflammatory factors and improve the antioxidant function of the body.

Reishi mushroom (*Ganoderma lucidum*) has the effects of invigorating qi, calming nerves, and relieving coughs and asthma. Cui et al. [68] adopted two steps: carbonization and activation. Firstly, *Ganoderma lucidum* was carbonized at 500 °C for 1.5 h. After carbonization, the powder was mixed with potassium hydroxide and activated for 1.5 h to prepare erythroid nitrogen and oxygen self-doped porous biomass carbon (GL800) derived from *Ganoderma lucidum* waste with high graphitization degree. The prepared GL800 shows a relatively high specific surface area and a porous structure similar to red blood cells, which provides sufficient space for accommodating active sites and facilitates electrolyte penetration. It has high application potential in energy storage, environmental restoration, wearable electronic equipment, biomedicine, and other fields.

### 3.4. Microwave-Assisted Method

The conventional synthesis methods introduced above usually involve high temperature conditions, and there are defects such as expensive heating equipment or high energy consumption, which is not in line with the core concept of green chemistry. With the increasing concern of environmental sustainability, it is hoped to develop greener methods to prepare CDs. The microwave-assisted method is a method to carbonize MFH directly into MFH-CDs under microwave radiation. The principle of microwave treatment is to realize the violent movement and mutual friction of drug molecules through microwave. The microwave method is an effective technology with great potential in the mass production of fluorescent CDs. An economical and simple microwave method can synthesize fluorescent CDs in a few minutes.

Lily (*Lilium brownii* var. *viridulum Baker*) has the functions of moistening the lungs, relieving coughs, clearing away heat, and calming nerves and diuresis. Gu et al. [64] used it as a carbon precursor and synthesized LB-CD with the microwave-assisted method. The synthesized LB-CD has high stability and fluorescence in an aqueous solution, and the quantum yield (QY) is 17.6%. Based on the fluorescence quenching stimulated by Cu^2+^ ions, LB-CDs were used as a fluorescence sensor for the detection of Cu^2+^ ions. In addition, lb-cd has low cytotoxicity and is suitable for fluorescence analysis and cell imaging.

Mint (*Mentha haplocalyx*) has the functions of dispelling wind and heat, clearing the head, relieving a sore throat, penetrating rashes, soothing the liver, and promoting qi circulation. Architha et al. [50] successfully synthesized blue fluorescent CDs (M-CDs) with a particle size of about 2.43 nm from mint with the microwave method. M-CDs with a uniform appearance and a controllable quality can be popularized in production because of its simple operation, energy saving, and high efficiency.

Ginkgo biloba Linn is flat, sweet, and bitter, and has little toxicity. It enters the lung and kidney meridians, and it has the effects of reducing blood viscosity, lowering blood pressure, eliminating phlegm, relieving a cough, and lowering cholesterol. Li et al. [79] used ginkgo fruit as the only carbon source, and synthesized two nitrogen-doped CDs (M-N-CDs/H-) by microwave-assisted and hydrothermal methods, respectively. Microwave-assisted synthesis time (5–15 min) is much shorter than that of the hydrothermal method (8–16 h), and its particle size is smaller (2.82 nm) than that of hydrothermal method (3.81 nm). However, as far as luminescence characteristics are concerned, although both N-CDs show typical absorption and PL spectra, the fluorescence intensity of H-N-CDs synthesized by hydrothermal method is much higher than that of M-N-CDs synthesized by microwave radiation. In addition, the QY and fluorescence lifetime of H-N-CDs are also longer than that of M-N-CDs. To sum up, microwave synthesis may be better than hydrothermal and pyrolysis methods in terms of preparation time and efficiency. Although the microwave method has the above advantages, it is still rare in the synthesis and application of MFH-CDs.

### 3.5. Other Methods

In addition to the above common methods, there are some methods that are not often used to prepare carbon dots in traditional Chinese medicine. For example, Gudimella et al. [69] grind papaya leaves and dilute them with water and then filter them with Whitman filter paper (nano-pore size). The filtrate was placed on a sand bath and then stirred at 180 °C for 24 h to obtain blue fluorescent CDs, which have good free radical scavenging activity, antioxidant activity, and anti-inflammatory activity in vitro.

*Lonicera japonica* Thunb. enters the lung, heart, and stomach meridians, and has the effects of clearing away heat and toxic materials, dispersing wind and heat, cooling the blood, and stopping dysentery. Gao et al. [75] prepared a honeysuckle extract/attapulgite/chitosan composite membrane containing natural carbon dots with the mechanical milling method. The composite membrane obtained has good mechanical properties, antibacterial properties, and antioxidant properties, and the composite membrane has an excellent antibacterial effect on *Escherichia coli* and *Staphylococcus aureus* and blue fluorescence performance.

## 4. Application of MFH-CDs

**Figure 5:**
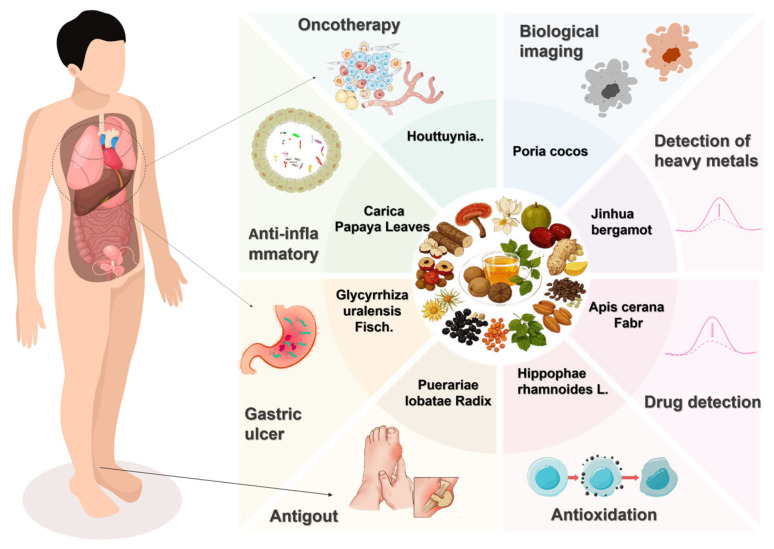
Application of MFH-CDs in medical treatment and environmental detection.

Traditional Chinese Medicine Carbon Dots (TCM-CDs) is considered to be the most promising fluorescent nanomaterial because of its excellent optical properties, photobleaching resistance, high water solubility, and low cytotoxicity. Compared with traditional Chinese medicine, the homologous substances of medicine and food are widely available and have been verified by long-term practice, which has higher safety and is more easily accepted by the public. In addition, the homologous materials of medicine and food usually come from natural food and edible plants, etc., which are non-toxic and have natural characteristics, so MFH-CDs have more advantages in biocompatibility and human adaptability than TCM-CDs. At present, the literature reports that MFH-CDs have applications in disease treatment [18,24,32,33,58,80,81], fluorescent probes [21,23,41,56,57,77,82,83,84,85,86,87,88,89,90,91], biological imaging [50,80,92,93,94,95], nanocatalysis [96,97,98], and other fields. The following will mainly introduce the application of these aspects (Figure 5).

### 4.1. Disease Treatment

#### 4.1.1. Anti-Gout and Anti-Inflammation

Abnormal metabolism of purine nucleotides in vivo is the main cause of gout disease, and xanthine oxidase (XOD) is the key enzyme for hypoxanthine transformation. Allopurinol is one of the main drugs to treat gout, and XOD can usually be used as an index to judge whether MFH-CDs has anti-gout activity. Common therapeutic drugs for gout have side effects. To study the anti-arthritis effect of MFH-CDs, the hyperuricemia model in rats is often induced by hypoxanthine, and its anti-uric acid ability is explored according to the XOD activity in the serum and livers of rats, and the influence on the arthritis model induced by sodium urate is observed, including the level of inflammatory factors and the histological changes in ankle inflammation.

*Aurantii Fructus Immaturus* (AFI), which originated from citrus plants, belongs to the spleen, stomach, and large intestine meridian, and its main functions are to break qi, eliminate food stagnation, resolve phlegm, and disperse swelling. Wang et al. [33] synthesized CDs (AFIC-CDs) from AFI and found that AFIC-CDs can inhibit the XOD activity in the serum and livers of hyperuricemia rats, reduce the content of inflammatory factors, and alleviate the pathological injury of gouty arthritis. At present, the treatment effect of hyperuricemia is not good, so it is very important to find a long-term and safe method to reduce uric acid and treat gouty arthritis. Related research also studies the anti-uric acid ability of XOD activity in the serum and livers of hyperuricemia rats and observes the changes related to inflammation.

*Puerariae Lobatae Radix* (PLR) is the root tuber of Leguminosae pueraria lobata or *Pueraria lobata*. It is a common and widely used medicine and food homologous plant, which contains a lot of carbon, nitrogen, and oxygen elements, and is an excellent material for preparing CDs. It was recorded in the medical book *Taiping Shenghui Fang* that purslane pueraria tea, a dietetic prescription, can reduce acid and induce diuresis. Wang et al. [32] synthesized *Pueraria lobata* carbon dots (PLR-CDs) from *Pueraria lobata* and found that PLR-CDS can not only reduce uric acid level by inhibiting XOD activity, but also improve ankle swelling and synovitis injury in rats with acute gouty arthritis, thus achieving anti-inflammatory activity.

#### 4.1.2. Treatment of Gastric Ulcer

*Glycyrrhiza uralensis* (GRR) is a perennial herb of Glycyrrhiza in Leguminosae, and it is one of the most commonly used Chinese herbal medicines to treat gastric ulcers. Gastric ulcer is a common chronic disease with a long course and easy recurrence, and the incidence rate is high among alcoholics [80]. Moreover, the experimental study also confirmed that glycyrrhizic acid in *Glycyrrhiza uralensis* Fisch has a good anti-ulcer effect, which can promote the secretion of mucus and bicarbonate in gastric mucosa and enhance the barrier function of gastric mucosa. Mucus can prevent gastric acid and pepsin from eroding gastric mucosa, thus achieving therapeutic effect. Bennett’s experiment on rats with gastric mucosal injury [81] showed that *Glycyrrhiza uralensis* Fisch has anti-ulcer activity, and further research by Ishii found that its mechanism may be related to its ability to reduce gastrin release. GRR-CDs prepared by Liu et al. [24] with licorice as the precursor by one-step pyrolysis may reduce the oxidative damage caused by alcohol to gastric mucosa and gastric tissue, which can be proved by the recovery of malondialdehyde, superoxide dismutase, and nitric oxide expression levels in the serum and tissues of mice. The results show that GRR-CDs have obvious anti-ulcer effects.

#### 4.1.3. Tumor Treatment

In the field of cancer prevention and treatment, ginger is the fresh rhizome of *Zingiber officinale* Rosc., a perennial herb of Zingiberaceae. Because of its antioxidant, antibacterial and carcinogenic properties, in the theory of traditional Chinese medicine, ginger is pungent in taste and warm in nature, and enters the lungs, spleen, and stomach meridians. It has the effects of relieving exterior syndrome, dispelling cold and warming, stopping vomiting, resolving phlegm, and relieving coughs, and relieving fish and crab poisoning. It can be used as a traditional medicinal material. Li et al. [18] used ginger as a raw material to synthesize CDs. Through the in vitro activity study of five different cell lines, it was found that it could induce the intracellular ROS level by up regulating the expression of *p53* gene, and it had obvious cytotoxicity to HepG2 cells at higher concentrations. In vivo studies also found that CDs can stay in tumor site through enhanced permeability and retention effect (EPR) of the body tumor, which can inhibit tumor growth and has obvious anti-liver cancer activity.

#### 4.1.4. Oxidation Resistance

As we all know, the occurrence of many diseases is inseparable from the excessive accumulation of free radicals, such as heart disease, diabetes, cancer, and so on. As a common therapeutic drug, antioxidants can effectively eliminate excessive accumulation of free radicals in the body, and proper intake of antioxidants is very important to prevent the occurrence of related diseases. Chinese herbal medicine and food contain a lot of antioxidant components, which are known as natural antioxidants.

*Hippophae rhamnoides* L. is a plant of Hippophae of Elaeagnaceae, which is sour, astringent, and warm. It has the effects of relieving coughs, resolving phlegm, invigorating the stomach, promoting digestion, promoting blood circulation, and removing blood stasis. It has a certain therapeutic effect on coughs with excessive phlegm, dyspepsia, dyspepsia, abdominal pain, blood stasis, and amenorrhea, and has good antioxidant activity, and can achieve antioxidant effect through multi-component and multi-target methods. Ma [58] deep eutectic solvents were used to extract antioxidant components from *Hippophae rhamnoides* L. Based on single factor experiments, the extraction process of antioxidant components from *Hippophae rhamnoides* L. was optimized by the response surface methodology. The antioxidant activity of *Hippophae rhamnoides* flavonoids was studied by analyzing the elimination rate of DPPH radical, OH radical, and ABTS radical. The results showed that *Hippophae rhamnoides* extract had strong antioxidant activity. The extracted *Hippophae rhamnoides* dregs are prepared into CDs, which improves the reuse of resources and turns waste into treasure.

### 4.2. Fluorescence Probe Detection

#### 4.2.1. Drug Detection

In order to ensure the quality of drugs, effective drug testing is needed to ensure the safety and reliability of drugs. Yang et al. [82] prepared water-soluble nitrogen-doped CDs (FCNs) by a simple and green one-step hydrothermal method with gardenia as the raw material, in which carbonization, surface functionalization, and doping occurred at the same time. It was found that the addition of metronidazole (MNZ) would cause the fluorescence quenching of FCNs, and with the increase in temperature, the fluorescence quenching constant gradually increased, that is, the increase in temperature was beneficial to fluorescence quenching and the quenching process. The linear range of MNZ concentration is 0.80~225.00 mol/L, and the detection limit is 279 nmol/L. It can be used for the analysis of biological samples.

Tiopronin (TPN) is a synthetic glycine derivative containing free sulfhydryl groups, which has active sulfhydryl groups and can protect the liver through various mechanisms [83,84]. Tiopronin can also reduce the side effects and anti-inflammatory and anti-allergic effects after chemotherapy. However, excessive use of tiopronin will lead to gastrointestinal discomfort, increased urinary protein content and taste failure [85], so it is particularly urgent to establish a method for detecting tiopronin.

*Apis cerana* Fabr. belongs to the lung, the spleen, and the large intestine meridian, and has the functions of invigorating middle energizer, moistening the lungs, relieving coughs, and relaxing the bowels. Li [57]. The fluorescent material CDs were prepared from honey with the hydrothermal method, and potassium permanganate was added to the solution of CDs to make them undergo a redox reaction, and the complex MnO_2_/CDs were obtained. Manganese dioxide made the carbon point lose fluorescence through the inner filter effect and static quenching. When tiopronin is added, the active sulfhydryl group in tiopronin reacts with manganese dioxide to reduce it to Mn^2+^, thus restoring the fluorescence of the carbon point. Therefore, the detection of tiopronin in tablets can be realized by using the “off–on” MnO_2_/CDs fluorescent probe.

Vitamin B_12_ is a kind of water-soluble vitamin containing cobalt, which plays an important role in the metabolism of substances in the body. Vitamin B_12_ deficiency or insufficient intake will lead to a series of diseases, such as pernicious anemia, mental depression, dysplasia of young children, spinal cord deformation, and so on [86]. In addition, excessive intake of vitamin B_12_ can also produce side effects, such as asthma, eczema, urticaria, facial edema, and other allergic symptoms. Therefore, it is very important to find a simple, fast, and efficient method for the determination of vitamin B_12_ content. Guo et al. [56] used saffron as a carbon source, and saffron synthesized blue fluorescent carbon dots with the one-step hydrothermal method. The results show that vitamin B_12_ has a remarkable quenching effect on the fluorescence of CD, and it has good practicability and universal applicability when applied to the analysis and detection of vitamin B_12_ in actual samples (vitamin B_12_ injection and vitamin B_12_ eye drops).

#### 4.2.2. Detection of Heavy Metal Ions

The rapid development of industrialization and human improper activities lead to a large number of toxic and harmful heavy metal ions being discharged into the water, soil, and other environmental media, causing serious pollution to the ecosystem [87]. Heavy metal ions existing in the environment have high toxicity and bioaccumulation effects, which can spread through the food chain and accumulate in organisms, causing serious damage to aquatic organisms and human health [88]. A large number of studies show that heavy metal pollution is related to a series of diseases such as neurodegenerative diseases, liver damage and renal dysfunction [89]. Therefore, the development of effective heavy metal detection methods is a key problem to be solved urgently in the field of pollutant detection.

Some MFH-CDs can directly react with heavy metal ions, causing electron transfer inside CDs and forming stable complexes, resulting in fluorescence quenching or enhancement. Sun et al. [21] used *Lycium barbarum* (LF) as a precursor to synthesize a new water-soluble LF-CDs, which was rich in hydroxyl groups on its surface and fluorescence quenched based on the internal filtration effect of LF-CDs and Fe^3+^. Among them, Hg^2+^ is one of the most toxic heavy metal ions, which can cause a series of diseases such as immune dysfunction, kidney damage, and cardiovascular damage [90]. Sun et al. [91] synthesized “on–off” type nitrogen-sulfur co-doped CDs from Gardeniae Fructus without any surface modification and passivation for the detection of Hg^2+^ and cysteine. Yu et al. used Jinhua bergamot as a carbon source to prepare green, low-cost, and water-soluble fluorescent carbon dots under hydrothermal conditions. The synthesized CDs have high selectivity, rapid response, low cost, and wide linear range in the detection of Hg^2+^ and Fe^3+^. In addition, many MFH-CDs including *Astragalus membranaceus* (Fisch.) Bunge [23], *Codonopsis Radix* [77], *Ziziphus jujuba* var. *spinosa* [41], etc. can be used to detect metal ions.

This is of great significance for the detection of drugs and heavy metal ions, ensuring the quality of pharmaceuticals and environmental safety. Taking the detection of metronidazole as an example, it can accurately detect the content of metronidazole in drugs, avoiding medical problems caused by inappropriate drug content. When it comes to detecting heavy metal ions, it can promptly identify heavy metal pollution in the environment, providing a scientific basis for environmental protection decision-making.

### 4.3. Biological Imaging

Based on the advantages of good photoluminescence, water dispersibility, narrow particle size distribution, and low cytotoxicity, researchers have successfully applied MFH-CDs to cell imaging in vitro and imaging in vivo [92,93].

#### 4.3.1. Cell Imaging

Cancer affects human health and kills millions of people every year. Therefore, advanced methods for early cancer diagnosis are becoming more and more important for preventing tumor development. Specially functionalized CDs can penetrate a variety of cancer cells and detect them more effectively through FL imaging. The special recognition mechanism mainly depends on the interaction between functional CDs and cancer cell surface groups. In cell imaging, the specific labeling of cancer cells is still a significant challenge. Therefore, it is more important to improve the specificity and targeting ability of fluorescent CDs for tracking cancer cells.

*Mentha haplocalyx* Briq. is an important medicinal and edible plant with antioxidant, anti-inflammatory, antibacterial, and anticancer activities. Because positively charged nanoparticles have stronger adhesion with negatively charged cell membranes and higher transfection efficiency, researchers used mint leaves as precursors and synthesized mint leaf CDs (P-CDs) by polyethyleneimine passivation to increase cell uptake [94]. Therefore, the uptake of human breast cancer (MCF-7) cells was further studied in P-CDs. It was found that the uptake of P-CDs was enhanced within 3 h after incubation. The small size enables P-CDs to be successfully absorbed into cells, thus providing fluorescence characteristics. At first, when there is no P-CDs fluorescence, only the red fluorescence of lysozyme tracker is produced. However, when the incubation time was extended to 3 h, bright green fluorescence could be seen. In addition, in the cancer environment, enhanced cell uptake is greatly affected, thus forming high-contrast cell markers. Therefore, the synthesized P-CDs can be used as an effective substitute for multifunctional imaging probes. Raveendran et al. [80] synthesized fluorescent CDs (M-CDs) from mint by using the green method. Considering the strong and stable photoluminescence characteristics of M-CDs, they tried to apply it to fluorescence imaging of HeLa cells. The hydrophilicity on the surface of M-CDs and the existence of functional groups enable them to easily internalize into cells through endocytosis and display multicolor fluorescence images at different excitation wavelengths. These results ensure the successful application of M-CDs as an effective probe for imaging.

Yellow fluorescent CD was prepared by hydrothermal carbonization of mint by Chang et al. [50], which has excellent stability, excellent biocompatibility, and ultra-low cytotoxicity. The CD can successfully target lysosomes with high co-localization coefficient (0.85) and respond to the fluctuation of Fe^3+^ in living cells. In addition, the obtained CD can be used for Escherichia coli imaging. In addition, the obtained CD is finally used to track the changes of Fe^3+^ in vivo system. The preliminary study shows that the synthesized CD can be used as an effective tool to detect Fe^3+^ in vitro and in vivo, and has a good application prospect in the detection of physiological and pathological diseases.

#### 4.3.2. In Vivo Imaging

The main research object of MFH-CDs in the field of biological imaging is cells, while biological imaging with microorganisms, protozoa, and plants as the research objects has not been studied for the time being. However, other biomass carbon dots are applied to in vivo imaging of mice. For example, Zhang et al. [95] used carbon dots from coffee bean shells as fluorescent probes to image mice with cervical cancer in vivo. The results showed that the obvious fluorescence signal could be observed in the tumor site 2 h after injection of carbon dots. After 24 h, the mice were dissected, and it was found that the carbon dots were mostly concentrated in the tumor and liver, and other parts were not affected. In addition, cervical cancer mice survived 6 days after the injection of carbon dots, which further proved that the carbon dots can be safely used for in vivo imaging.

### 4.4. Nanocatalysis

**Figure 6:**
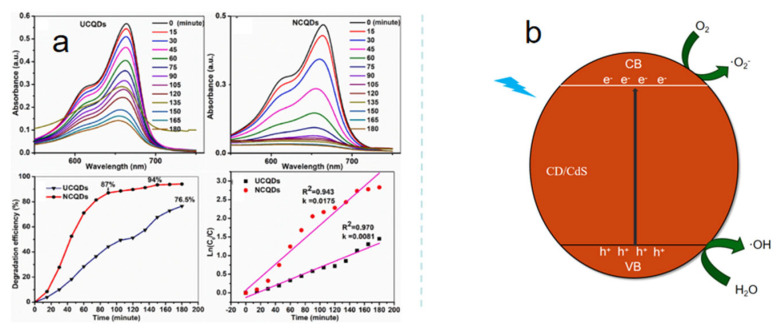
(**a**) Photocatalytic degradation of coconut shell carbon dots, (**b**) photocatalytic mechanism of neem leaf carbon dots (CD)-modified cadmium sulfide (CdS) nanomaterials.

Nanocatalysts refer to materials with sizes ranging from 1 to 100 nanometers, formed by the combination of nano-active components and a support. Its advantages are significant: a high specific surface area provides a large number of active sites, resulting in extremely high catalytic activity; the size and shape are controllable, allowing the precise guidance of specific reactions with strong selectivity; with proper preparation, it has good stability and a long lifespan [96]. Among them, foods that are both medicinal and edible usually contain some active substances that aid in catalysis. For example, coconut shells have certain medicinal values. It contains various active substances such as coconut shell phenol, coconut fatty acid, and flavonoids, which have antibacterial, antioxidant, and anti-inflammatory properties. Edakkaparamban and others synthesized coconut shell carbon dots as nanocatalysts, primarily for the degradation of methylene blue, a toxic and harmful dye pollutant [97]. Undoped (UCQDs) and nitrogen-doped (NCQDs) coconut shell carbon dots synthesized through a one-step hydrothermal method both exhibit photocatalytic activity under white light irradiation. Among them, NCQDs performed even better, achieving a degradation rate of 94% for methylene blue within 150 min, which is significantly higher than the 76.5% achieved by UCQDs (180 min). Moreover, NCQDs are less affected by pH and can stably degrade methylene blue within the pH range of 3 to 10, achieving a degradation efficiency of about 93% (Figure 6). This gives them great potential for application in wastewater treatment under different acidic and alkaline conditions.

The neem tree is sometimes referred to as the “village pharmacy”. It is a unique medicinal plant, and all its parts—leaves, flowers, seeds, fruit, roots, and bark—can be used for medicinal purposes. This tree is highly valued in Africa, especially in the Sahel region, and it is used for many different purposes. The leaves of the neem tree are used in traditional medicine to treat skin diseases, fevers, and some infectious diseases. Choudhary et al. [98] used neem leaf carbon dots (CD) to modify cadmium sulfide (CdS) to form a nanocatalyst, exploring its photocatalytic performance in degrading organic pollutants such as ciprofloxacin in water. CD/CdS can utilize light energy to produce reactive oxygen species, disrupting the molecular structure of pollutants. Research shows that compared to original CdS, CD/CdS has a higher degradation rate for ciprofloxacin, reaching approximately 75% (Figure 6). Moreover, under conditions of neutral pH, high catalyst loading, low feed concentration, higher temperature, and high lamp power, its degradation effect is even better. As nanocatalysts, food-grade carbon dots have demonstrated multifaceted application potential in the field of photocatalysis, achieving certain research results in both environmental pollution control and energy conversion. In the future, with the continuous in-depth research on performance optimization and mechanism of action, it is expected to achieve broader application fields.

## 5. Advantages of MFH-CDs

The biggest characteristic of materials that are both food and medicine is that they themselves are rich in various bioactive components, such as corn silk, which is rich in flavonoids, saponins, alkaloids, amino acids, and polysaccharides. Carbon dots prepared from food–medicine homologous materials retain the pharmacological activity of these medicinal materials, exhibiting multiple bioactivities such as immune regulation, anti-tumor, antioxidant, blood pressure reduction, and antibacterial properties without the need for additional surface passivation or doping [99]. In contrast, CQDs from other sources lack this characteristic and are more commonly used in areas such as photoelectrocatalysis. For example, Zhang et al. [100] used citric acid as a carbon source to prepare carbon quantum dots (CQDs) through a one-step hydrothermal method and synthesized layered mesoporous titanium dioxide (LM-TiO_2_) using an inorganic precipitation-gel sol method. Subsequently, CQDs and LM-TiO_2_ were mixed to obtain carbon quantum dot/layered mesoporous titanium dioxide composites (CQDs/LM-TiO_2_). After 24 min of irradiation, the degradation rate of 0.2 wt% CQDs/LM-TiO_2_ for methyl orange was 91.04%, which is significantly higher than that of pure TiO_2_ and LM-TiO_2_. This indicates that this type of CQDs itself has certain photocatalytic properties, and through the synergistic effect, it increases the light absorption range after combination, among other benefits.

MFH-CDs are derived from substances that are either edible or have medicinal value, and they usually possess good biocompatibility. Carbon dots prepared from medicinal and edible materials like hawthorn and red dates are less likely to cause immune reactions or toxicity in the body, giving them a greater advantage in biomedical fields such as bioimaging and drug delivery. In contrast, CQDs from other sources may have biocompatibility issues and require additional surface modifications to improve. Wang et al. [101] prepared sulfur and nitrogen co-doped carbon quantum dots (S, N-CQDs) through a one-pot hydrothermal treatment using disodium EDTA and sodium sulfide. Cell experiments indicate that N-CQDs have good biocompatibility, as they show no significant toxicity to both normal cell lines and cancer cells. Based on the good biocompatibility of S,N-CQDs, dopamine in actual serum samples was detected, and the results showed an excellent recovery rate. By doping ions to modify the surface, the electronic structure, crystal structure, and other properties of the material can be altered, thereby affecting its optical, electrical, catalytic, and detection performance.

At the same time, substances that are both food and medicine have undergone extensive safety verification through long-term consumption and medicinal use and are generally considered safe and reliable. The carbon dots prepared from these substances inherit this safety. Compared to some chemically synthesized or non-food-derived CQDs, they have obvious advantages when applied in fields such as food and medicine, where safety is of high importance, and can reduce potential safety risks.

## 6. Summary and Prospect

As a new carbon nano-material, CDs have excellent biological characteristics, sensitivity, photoluminescence, and stability, and are widely used in ion detection, biological imaging, and sensing. As a brand-new branch of CDs, MFH-CDs are green and safe and have been widely used in the above fields. The application of MFH-CDs in biology can provide new ideas for the future development of therapeutics. MFH-CDs also have some defects. For example, the most commonly used synthesis methods of CDs are the hydrothermal method and the microwave-assisted method, which lead to unstable fluorescence intensity and particle size of CDs. However, by optimizing reaction conditions (such as temperature, time, and raw material ratios), introducing microfluidic technology to precisely control the reaction environment, and performing post-synthesis screening of the synthesized CDs, the issues of instability in CDs’ fluorescence intensity and particle size can be effectively addressed, thereby enhancing the uniformity and performance of the products. In addition, different precursors of MFH-CDs synthesis have different pharmacological activities. In the process of high-temperature carbonization of Chinese medicinal materials, some effective components in Chinese medicinal materials may decompose or produce new effective components. At present, MFH-CDs have achieved initial results and progress in the treatment of diseases, but there are relatively few studies on the treatment mechanism and effective component analysis, which can be used as a direction of MFH-CDs research in the future. Secondly, although MFH-CDs show good safety in cell imaging and sensing in vivo, it is not clear whether it will accumulate in vivo and produce adverse reactions after long-term treatment. The essence of MFH-CDs are nanoparticles, so its metabolic pathway in vivo is also one of the challenges for future research. In a word, the application of MFH-CDs in the field of biology is still in the primary stage, and there is a wide development space and unlimited possibilities in the future. The precursors of MFH-CDs synthesis mostly come from plants but less are involved in animal drugs. In the later stage, we can explore and study animal-like MFH-CDs.

## Figures and Tables

**Table 1:** Precursors, synthesis methods, applications, and fluorescence properties of MFH-CDs.

Precursor	Synthetic Method	Application	Quantum Yield (%)	Size (nm)	Fluorescent Color	Ref.
*Ziziphus jujuba* var. *spinosa*	Hydrothermal	Fluorescence detection of Hg^2+^	16.9%	1.2 nm	Blue	[41]
*Hibiscus trionum* L.	Hydrothermal	Detecting ammonia vapor	2%	-	Blue	[22]
*Dimocarpus longana* Lour.	Hydrothermal	Antioxidant, anti-inflammatory and Fe^2+^ detection	15.2%	4.55 nm	Blue	[42]
*Ipomoea batatas*	Hydrothermal	Promote the repair of bone defect	4.44%	2–6 nm	Blue	[43]
*Crataegus pinnatifida* Bunge	Hydrothermal	Detection of chlortetracycline in pork samples	22.96%	3.17 nm	Blue	[44]
*Lycium chinense* Mill.	Hydrothermal	Ag^+^ detection	21.8%	3.0–6.5 nm	Green	[38]
*Lycium chinense* Mill.	Hydrothermal	Fe^3+^ detection	17.2%	2–5 nm	Blue	[21]
*Ganoderma lucidum*	Hydrothermal	Determination of copper ion	4.6% 2.6%	1.57–2.83 nm, 2.03–3.85 nm	Blue green	[45]
*Nelumbo nucifera*	Hydrothermal	Determination of acid phosphatase	8.6%	2.92 ± 0.30 nm	Green	[46]
*Chrysanthemum morifolium* Ramat.	Hydrothermal	Curcumin sensor, fluorescent ink	28.5%	1.7–3.3 nm	Blue	[47]
*Prunus persica*, *Carthamus tinctorius*	Hydrothermal	Improve the permeability of blood–brain barrier	3.84%	3.12 ± 0.95 nm	Blue	[48]
*Citrus reticulata*	Hydrothermal	Degradable dye	12.3%	2−7 nm	Blue	[49]
*Curcuma longa*	Hydrothermal	Antibiosis	-	2.6 nm	Blue	[17]
*Mentha haplocalyx*	Hydrothermal	Iron ion detection	6.9%	6.66 ± 0.83 nm	Yellow	[50]
*Astragalus membranaceus*	Hydrothermal	Fe^3+^ detection, biological imaging	35.6%	4.22 ± 0.23 nm	Blue	[23]
*Cornus officinalis*	Hydrothermal	Detection of p-nitrophenol and photocatalytic degradation of dyes	18.3%	3.53 nm	Green	[51]
*Eucommia ulmoides*	Hydrothermal	Detection of Al^3+^ in water	42.3%	3.55 ± 1.45 nm	Blue	[52]
*Dendrobium officinale*	Hydrothermal	Detection of MAP and OAP in real water samples	-	16–24 nm	-	[53]
*Poria cocos*	Hydrothermal	Cell imaging, free radical scavenging and pH sensing	1%	1.5–4.5 nm	Blue	[31]
*Zingiber officinale*	Hydrothermal	Inhibition of liver cancer cells	-	4.3 ± 0.8 nm	Blue	[18]
*Citrus medica* var. *sarcodactylis*	Hydrothermal	Detect Hg^2+^ and Fe^3+^	50.78%	10 nm	Blue	[19]
*Cassia obtusifolia*	Hydrothermal	Photocatalytic degradation of methylene blue	-	1.23 nm	Kelly	[54]
*Trigonella foenum-graecum*, *Syzygium aromaticum*, *Cuminum cyminum*	Hydrothermal	Determination of sunset yellow in drinks by yellow–green color	-	5–10 nm	-	[55]
*Crocus sativus* L.	Hydrothermal	VB_12_ detection	-	4.1 ± 0.3 nm	Blue	[56]
*Mel*	Hydrothermal	Detection of tiopronin		2.02 ± 0.11 nm	Blue	[57]
*Hippophae rhamnoides* L.	Hydrothermal	Cell imaging	-	3–5 nm	Blue	[58]
*Semen armeniacae amarum*	Hydrothermal	Cell imaging and Fe^3+^ biosensing	-	5 ±0.6 nm	Blue	[59]
*Zanthoxylum bungeanum* Maxim.	Hydrothermal	Fluorescent anti-counterfeiting ink	13.1%, 11.2% and 9.8%	3.5 nm, 4.0 nm and 7.5 nm	Blue, green and red	[60]
*Gardenia jasminoides*	Hydrothermal	Protective effect of cell oxidative damage		4.8 ± 0.52 nm	Blue	[61]
*Taraxacum mongolicum*	Solvothermal	Fluorescence detection of Fe^3+^, Pb^2+^ and Sn^4+^	43.8%, 32.9%, 48.1%	3.5 nm, 2.6 nm, 2.3 nm	Blue, green, orange	[27]
*Chaenomeles sinensis*		NIR-stimulated photochemotherapy and antibacterial activity	12.34%	5−9 nm	Blue	[29]
*Houttuynia cordata*, *Lonicera japonica*, *Artemisia argyi*, *Perilla frutescens*, *Taxus chinensis*	Solvothermal	Tumor imaging and therapy	0.59%, 0.42%, 1.13%, 0.55%, 1.01%	-	Red	[62]
*Piper nigrum*	Solvothermal	Determination and ascorbic acid imaging	10.25%	4.7 nm	-	[63]
*Lilium brownii* var. *viridulum*	Microwave	Cu^2+^ ion detection	17.6%	1.34–5.23 nm	-	[64]
*Dimocarpus longan*	Microwave	Detection of metronidazole	-	2.8–4.2 nm	-	[65]
*Chaenomeles sinensis*	Microwave	Fe^3+^ ion detection	9.7%	1.45 nm	Blue	[66]
*Chaenomeles sinensis*	Microwave	Detection and degradation of tetracycline hydrochloride in water	10.05%	2–6.5 nm	Blue	[67]
*Platycodon grandiflorus*	Pyrolysis	Treat hyperbilirubinemia	-	1.2–3.6 nm	-	[26]
*Ganoderma lucidum*	Pyrolysis	Electrochemistry				[68]
*Glycyrrhiza uralensis*	Pyrolysis	Antigastric ulcer effect	2.51%	1–5 nm	-	[24]
*Pueraria lobata*	Pyrolysis	Treat gout	3.2%	3.0–10.0 nm	-	[32]
*Chaenomeles sinensis*	Sand bath	Antioxidant and anti-inflammatory effects	-	-	Blue	[69]
*Lonicera japonica*	Mechanical milling	Food packaging	-	-	Blue	[70]
*Siraitia grosvenorii*	-	-	0.05%	9.0 nm	-	[71]
